# Longitudinal study revealing motor, cognitive and behavioral decline in a transgenic minipig model of Huntington's disease

**DOI:** 10.1242/dmm.041293

**Published:** 2019-12-12

**Authors:** Monika Baxa, Bozena Levinska, Monika Skrivankova, Matous Pokorny, Jana Juhasova, Jiri Klima, Jiri Klempir, Jan Motlík, Stefan Juhas, Zdenka Ellederova

**Affiliations:** 1 Laboratory of Cell Regeneration and Plasticity, Institute of Animal Physiology and Genetics, Czech Academy of Sciences, 277 21 Libechov, Czech Republic; 2 Department of Cell Biology, Faculty of Science, Charles University in Prague, 128 00 Prague, Czech Republic; 3 Department of Circuit Theory, Faculty of Electrical Engineering, Czech Technical University in Prague, 166 27 Prague, Czech Republic; 4 Department of Neurology and Centre of Clinical Neuroscience, First Faculty of Medicine, Charles University in Prague and General University Hospital in Prague, 128 21 Prague, Czech Republic

**Keywords:** Huntington's disease, Large animal model, Phenotyping, Motor, Cognitive and behavioral studies

## Abstract

Huntington's disease (HD) is an inherited devastating neurodegenerative disease with no known cure to date. Several therapeutic treatments for HD are in development, but their safety, tolerability and efficacy need to be tested before translation to bedside. The monogenetic nature of this disorder has enabled the generation of transgenic animal models carrying a mutant huntingtin (mHTT) gene causing HD. A large animal model reflecting disease progression in humans would be beneficial for testing the potential therapeutic approaches. Progression of the motor, cognitive and behavioral phenotype was monitored in transgenic Huntington's disease minipigs (TgHD) expressing the N-terminal part of human mHTT. New tests were established to investigate physical activity by telemetry, and to explore the stress-induced behavioral and cognitive changes in minipigs. The longitudinal study revealed significant differences between 6- to 8-year-old TgHD animals and their wild-type (WT) controls in a majority of the tests. The telemetric study showed increased physical activity of 4.6- to 6.5-year-old TgHD boars compared to their WT counterparts during the lunch period as well as in the afternoon. Our phenotypic study indicates progression in adult TgHD minipigs and therefore this model could be suitable for longstanding preclinical studies of HD.

This article has an associated First Person interview with the first author of the paper.

## Abstract (precis)

**Summary:** The transgenic minipig model of Huntington's disease demonstrates a slow-progressing motor, cognitive and behavioral phenotype with later onset in adulthood.

## INTRODUCTION

The clinical symptoms of neurodegenerative Huntington's disease (HD) are motor, cognitive and behavioral impairments manifesting typically in the mid-30s (Nance, 1998). Motor problems include involuntary chorea-like movement, poor balance and disturbed fine motor skills (Beighton and Hayden, 1981; David et al., 1987). The most prominent cognitive symptoms include impaired judgment; the inability to initiate, sustain attention and complete a task; and difficulty with tasks requiring flexibility or speed (Brandt et al., 1984; Lai et al., 2018). Behavioral disturbances include anxiety, depression, irritability, obsessiveness, and impulsive and aggressive behavior interchanging with apathy (Eddy et al., 2016). HD is induced by abnormal polyglutamine elongation of the gene encoding the huntingtin protein (HTT). Although the cause of HD was discovered in 1993, the disease remains incurable and needs a suitable model for testing potential therapies. Large animal models can provide better preclinical outcomes – including safety, biodistribution, longitudinal assessment and efficacy of novel therapeutic approaches – compared to rodents (Howland and Munoz-Sanjuan, 2014). Therefore, large-animal models, such as non-human primates (Kocerha et al., 2013; Yang et al., 2008), sheep (Jacobsen et al., 2010), and pigs or minipigs (Baxa et al., 2013; Uchida et al., 2001; Yan et al., 2018; Yang et al., 2010), have been generated. Among these, minipigs represent a good economical and ethical choice (Morton and Howland, 2013). Their brain is quite large and similarly structured to that of humans. In addition, their similar metabolism, body weight, longevity of 15-20 years and high reproduction make them suitable for translational research (Vodička et al., 2005).

In 2009, a transgenic minipig model for HD (TgHD) expressing the N-terminal part of human mutated huntingtin (mHTT; 548 amino acids, 124 Q) was generated (Baxa et al., 2013). Generally, all tissues isolated from TgHD minipigs from different generations express human mHTT as well as endogenous HTT (Macakova et al., 2016; Vidinská et al., 2018). Broad phenotypic studies of TgHD minipigs compared to their wild-type (WT) siblings are ongoing. The phenotype development of the model was rather slow, and the first clear phenotype preceding the neurodegenerative one was sperm and testicular degeneration linked with mitochondria metabolism and glycolytic impairment starting at 13 months of age (Krizova et al., 2017; Macakova et al., 2016). However, reduction of DARPP32 (also known as PPP1R1B), a marker of proper function of spiny neurons, has been detected from 16 to 70 months (Baxa et al., 2013; Vidinská et al., 2018; Ardan et al., 2020). Also, other markers of neurodegeneration, such as activation of microglia and demyelination of white matter, together with mHTT gradual fragmentation, were revealed at 24 months (Vidinská et al., 2018). Mitochondrial DNA damage and a marker of metabolic alteration were detected at 48 months (Askeland et al., 2018). Furthermore, perturbed mitochondrial function was detected in TgHD minipig muscle tissue starting at 36 months, before alteration in muscle mitochondria ultrastructure and first locomotor decline at the age of 48 months (Askeland et al., 2018; Rodinova et al., 2019). TgHD-genotype-specific significant cellular loss in the striatum and cortex, together with inclusions in the axons of some neurons, were detected at 60-70 months (5-5.8 years) (Ardan et al., 2020).

This study aimed to longitudinally monitor the motor, cognitive and behavioral phenotype of TgHD minipigs up to 8 years. Gait, hurdle and startbox back-and-forth tests using TgHD minipigs were established in the George-Huntington-Institute in Münster (Schramke et al., 2016; Schuldenzucker et al., 2017). Activity was measured by a telemetric system adapted for minipigs, and cover and skittle toys for cognitive measures, and a balance beam and seesaw for stress-induced tests, were established by us. Within the 4 subsequent years, 4- to 7.9-year-old TgHD minipigs (*n*=8) and their WT controls (*n*=10) were monitored using motor, cognitive and behavioral tests; and, simultaneously, the physical activity of TgHD boars (*n*=6) and their WT siblings (*n*=6) at 2.5-6.5 years was investigated by telemetry.

## RESULTS

### Motor impairment

**Fig. 1.**
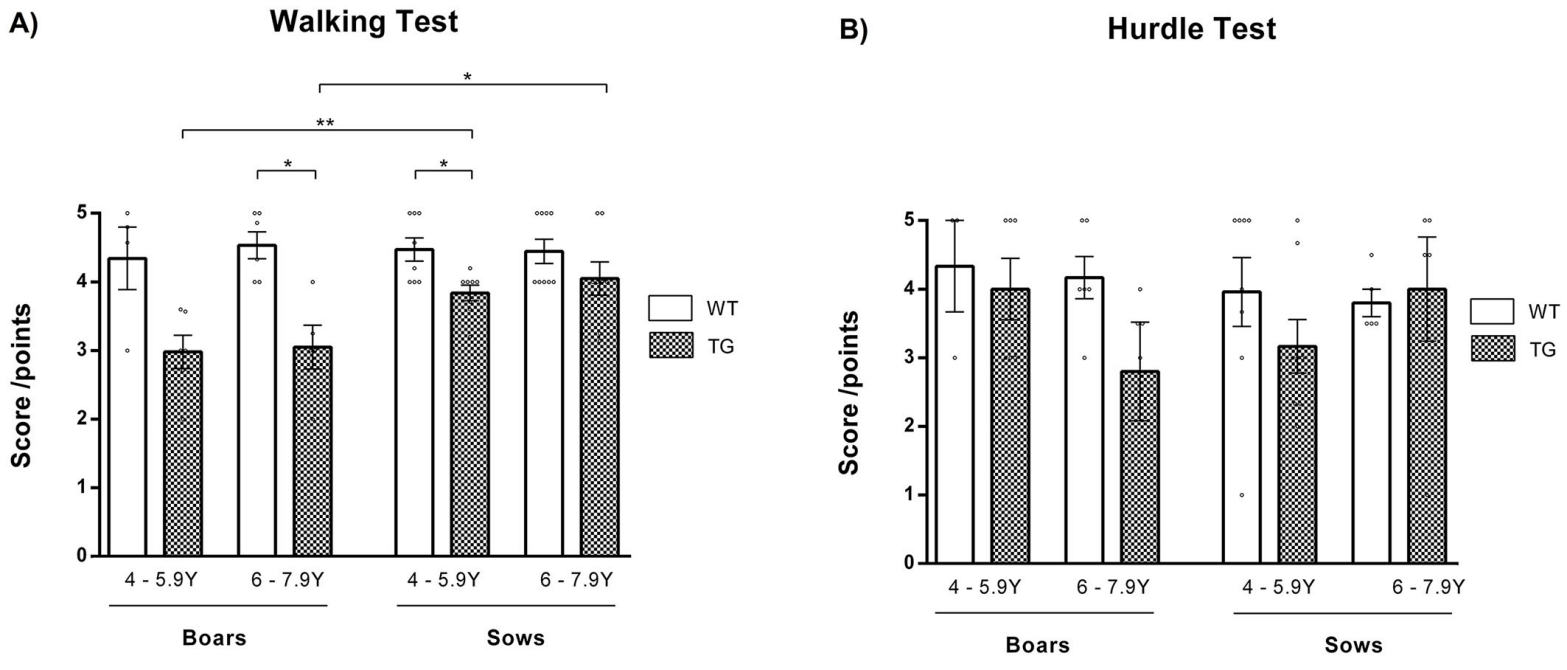
**Motor impairment.** (A) Walking test. Impairment in gait was observed in TgHD animals in comparison to their WT controls. The difference was significant in 6- to 7.9-year-old TgHD boars (*P*=0.013) and 4- to 5.9-year-old TgHD sows (*P*=0.015). The decline was more obvious in TgHD boars than in TgHD sows. Clear sex-related differences were noticed between TgHD boars and TgHD sows of matching ages (*P*=0.003 and *P*=0.024, respectively). WT boars 4-5.9 years *n*=4, WT boars 6-7.9 years *n*=6, TgHD boars 4-5.9 years *n*=6, TgHD boars 6-7.9 years *n*=5, WT sows 4-5.9 years *n*=8, WT sows 6-7.9 years *n*=9, TgHD sows 4-5.9 years *n*=8, TgHD sows 6-7.9 years *n*=8. (B) Hurdle test. The ability to cross a barrier declined in TgHD boars with age. TgHD sows obtained a worse score at the age of 4-5.9 years, but a better score at the age of 6-7.9 years, in comparison to their age-matched WT controls. Scores were lower in TgHD boars than in TgHD sows at the age of 6-7.9 years, but this difference was not significant. WT boars 4-5.9 years *n*=3, WT boars 6-7.9 years *n*=6, TgHD boars 4-5.9 years *n*=6, TgHD boars 6-7.9 years *n*=5, WT sows 4-5.9 years *n*=8, WT sows 6-7.9 years *n*=5, TgHD sows 4-5.9 years *n*=8, TgHD sows 6-7.9 years *n*=5. *P≤0.05, **P≤0.01.

As the clearest clinical hallmark of HD is movement detriment, unsteady gait and lack of coordination in patients (Caron et al., 1993; Vuong et al., 2018), the gait of minipigs was examined. The walking test was performed on a flat dry floor; in the hurdle test, the minipig was challenged with an obstacle. The walking test (Fig. 1A) revealed impairment in TgHD animals. The decrease in walking score was significant in TgHD boars at 6-7.9 years (*P*=0.013), and in TgHD sows at 4-5.9 years (*P*=0.015), compared with their age-matched WT controls. More evident changes in walking were observed among TgHD boars. TgHD animals exhibited different movement of the hind legs (Movie 1). Significant sex-related differences were observed between TgHD boars and TgHD sows at younger (4-5.9 years) and older (6-7.9 years) age (*P*=0.003 and *P*=0.024, respectively).

At the age of 4-5.9 years, both TgHD boars and TgHD sows had lower scores in the hurdle test (Fig. 1B) than their WT controls, but these differences were non-significant. Sex-related differences in test performance were observed between boars and sows with aging; TgHD boars showed continuing deterioration within aging in comparison to WT animals, whereas TgHD sows showed better test scores at 6-7.9 years.

Corrective responses to externally generated force pulses are disturbed in HD patients (Smith et al., 2000); therefore, the pull-back test (Movie 2) was introduced in minipigs. This test simulated unexpected disruption of an animal's balance. When 6-year-old TgHD boars were shoved in the haunch area, they lost their balance, and their hind legs slipped and moved in the opposite direction to which the sudden pulse occurred. Their front legs reached a wider position in an effort to maintain stability and not fall. Control WT animals quickly regained balance with no problems concerning their legs.

**Fig. 2.**
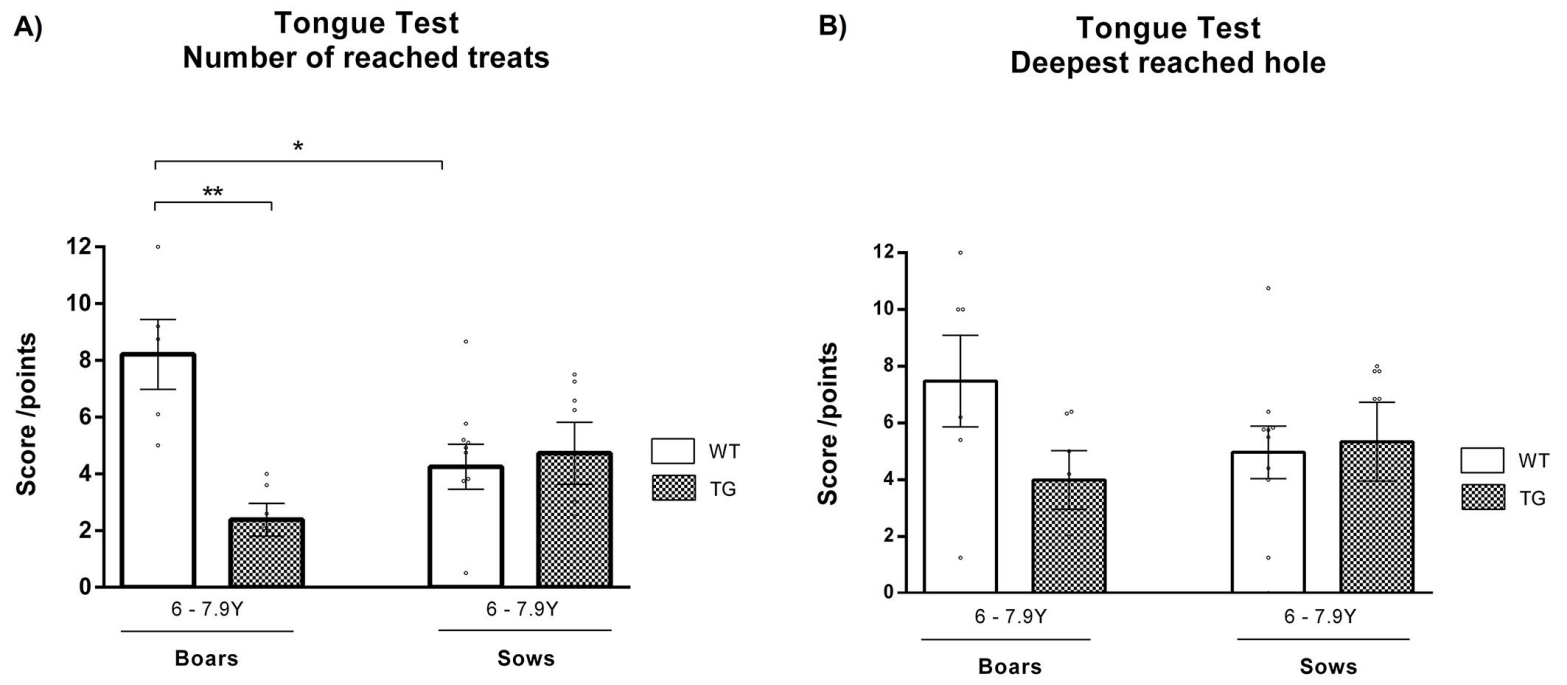
**Tongue protrusion persistence.** (A) In the number-of-reached-treats test, a significantly (*P*=0.004) lower score was observed in TgHD boars than in WT boars. (B) The deepest-hole test revealed a lower score in TgHD boars, and a similar score in TgHD sows, in comparison to their age-matched WT controls. WT boars 6-7.9 years *n*=5, TgHD boars 6-7.9 years *n*=6, WT sows 6-7.9 years *n*=10, TgHD sows 6-7.9 years *n*=7. *P≤0.05, **P≤0.01.

Next, fine motor skills were investigated. Motor impersistence manifesting as difficulty to keep the tongue fully protruded for a few seconds has been observed in HD patients (Huntington Study Group 1996); therefore, a tongue test was introduced to monitor the protrusion persistence of the animals. The number-of-reached-treats test (Fig. 2A) showed significantly (*P*=0.004) decreased score in TgHD boars, but very similar score in TgHD sows, in comparison to their WT controls. However, the deepest-reached-hole test (Fig. 2B) revealed non-significantly decreased score in TgHD boars, and almost the same score in TgHD sows, compared to WT.

### Cognitive changes

**Fig. 3.**
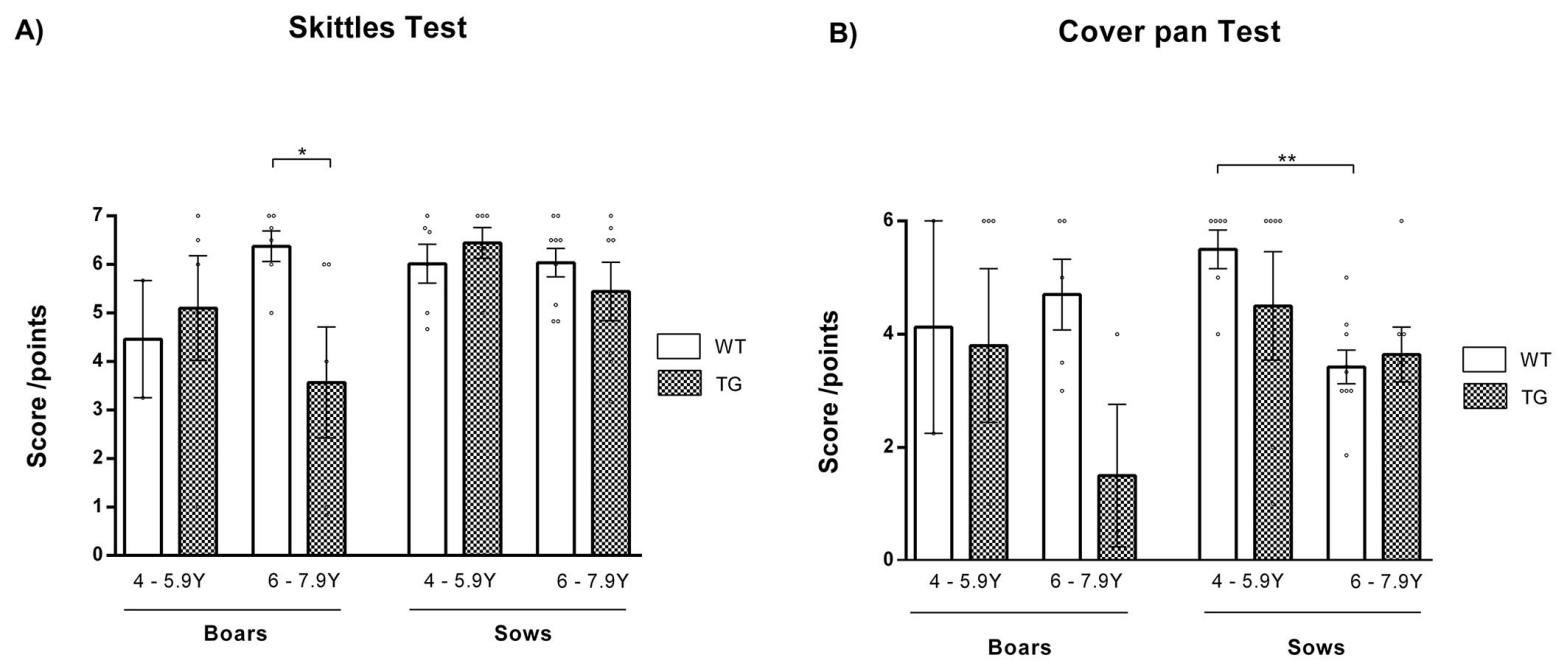
**Cognitive changes.** (A) In the skittles test, a significantly (*P*=0.03) decreased score was detected in TgHD boars compared to WT boars at the age of 6-7.9 years, but scores were similar among all sows. WT boars 4-5.9 years *n*=2, WT boars 6-7.9 years *n*=6, TgHD boars 4-5.9 years *n*=5, TgHD boars 6-7.9 years *n*=5, WT sows 4-5.9 years *n*=6, WT sows 6-7.9 years *n*=9, TgHD sows 4-5.9 years *n*=6, TgHD sows 6-7.9 years *n*=7. (B) In the cover pan test, scores were lower in 6- to 7.9-year-old TgHD boars than in their WT controls, but this difference was not statistically significant. Impairment in the ability to perform the test with age was observed. WT boars 4-5.9 years *n*=2, WT boars 6-7.9 years *n*=5, TgHD boars 4-5.9 years *n*=5, TgHD boars 6-7.9 years *n*=3, WT sows 4-5.9 years *n*=6, WT sows 6-7.9 years *n*=9, TgHD sows 4-5.9 years *n*=6, TgHD sows 6-7.9 years *n*=7. *P≤0.05, **P≤0.01.

Cognitive deficits emerge in HD and ordinarily influence ability to learn tasks, spatial navigation and working memory. Hence, new tests for monitoring these functions in minipigs were developed in this study. The skittles test consisted of a pan with seven holes concealed by seven different skittles. Pigs were expected to flip as many skittles as possible to find treats hidden under the skittles. The cover pan test consisted of a pan with six holes closed by six different movable covers. Animals were expected to move the cover to reach the treat hidden underneath it. In the skittles test (Fig. 3A), a significantly (*P*=0.03) lower score was observed in 6- to 7.9-year-old TgHD boars than in their age-matched WT controls, whereas TgHD females showed similar results to WT females. Similarly, 6- to 7.9-year-old TgHD males obtained a lower score than their WT counterparts in the cover pan test (Fig. 3B), whereas the scores of TgHD and WT females were comparable. A decline in score was observed in TgHD animals with aging.

### Decline in stress-induced performance

**Fig. 4.**
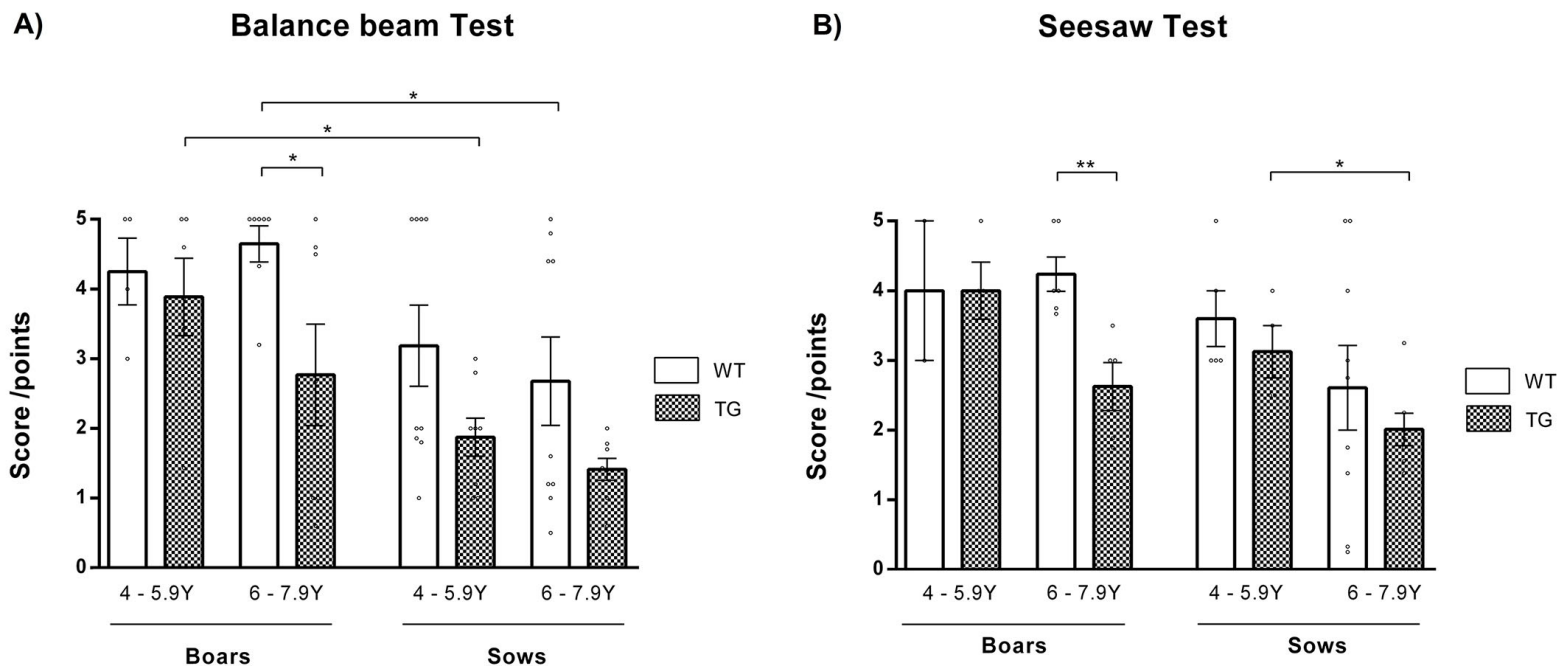
**Decline in stress-induced performance.** (A) In the balance beam test, significant (*P*=0.033) deterioration was observed in TgHD boars compared to their WT controls at the age of 6-7.9 years. Both TgHD and WT sows had lower scores than boars at older age (*P*=0.012 for TgHD at 4-5.9 years; *P*=0.021 for WT at 6-7.9 years). The ability to pass the assessment decreased with age in all TgHD animals. WT boars 4-5.9 years *n*=4, WT boars 6-7.9 years *n*=7, TgHD boars 4-5.9 years *n*=6, TgHD boars 6-7.9 years *n*=7, WT sows 4-5.9 years *n*=9, WT sows 6-7.9 years *n*=9, TgHD sows 4-5.9 years *n*=8, TgHD sows 6-7.9 years *n*=8. (B) In the seesaw test, TgHD boars obtained a significantly (*P*=0.004) lower score than their WT controls at 6-7.9 years. The attained score decreased with age in all TgHD animals, significantly (*P*=0.024) in TgHD sows. WT boars 4-5.9 years *n*=2, WT boars 6-7.9 years *n*=6, TgHD boars 4-5.9 years *n*=4, TgHD boars 6-7.9 years *n*=5, WT sows 4-5.9 years *n*=5, WT sows 6-7.9 years *n*=9, TgHD sows 4-5.9 years *n*=4, TgHD sows 6-7.9 years *n*=7. *P≤0.05, **P≤0.01.

HD patients have difficulties in coping with stressful situations. Therefore, tests inducing stress were investigated; namely, a balance beam test and a seesaw test. The balance beam test (Fig. 4A) was previously applied in a pig model of ataxia telangiectasia (Beraldi et al., 2015). The seesaw test (Fig. 4B) was newly established in this study. Both tests showed a lower score obtained by TgHD animals in comparison to their age-matched WT controls. Significant differences were detected between 6- to 7.9-year-old TgHD and WT boars in the balance beam and seesaw tests (*P*=0.033 and *P*=0.004, respectively). TgHD animals were not willing to walk on the balance beam (Movie 3). TgHD sows showed significant age-dependent impairment in performance in the seesaw test (*P*=0.024).

### Altered physical activity

**Fig. 5.**
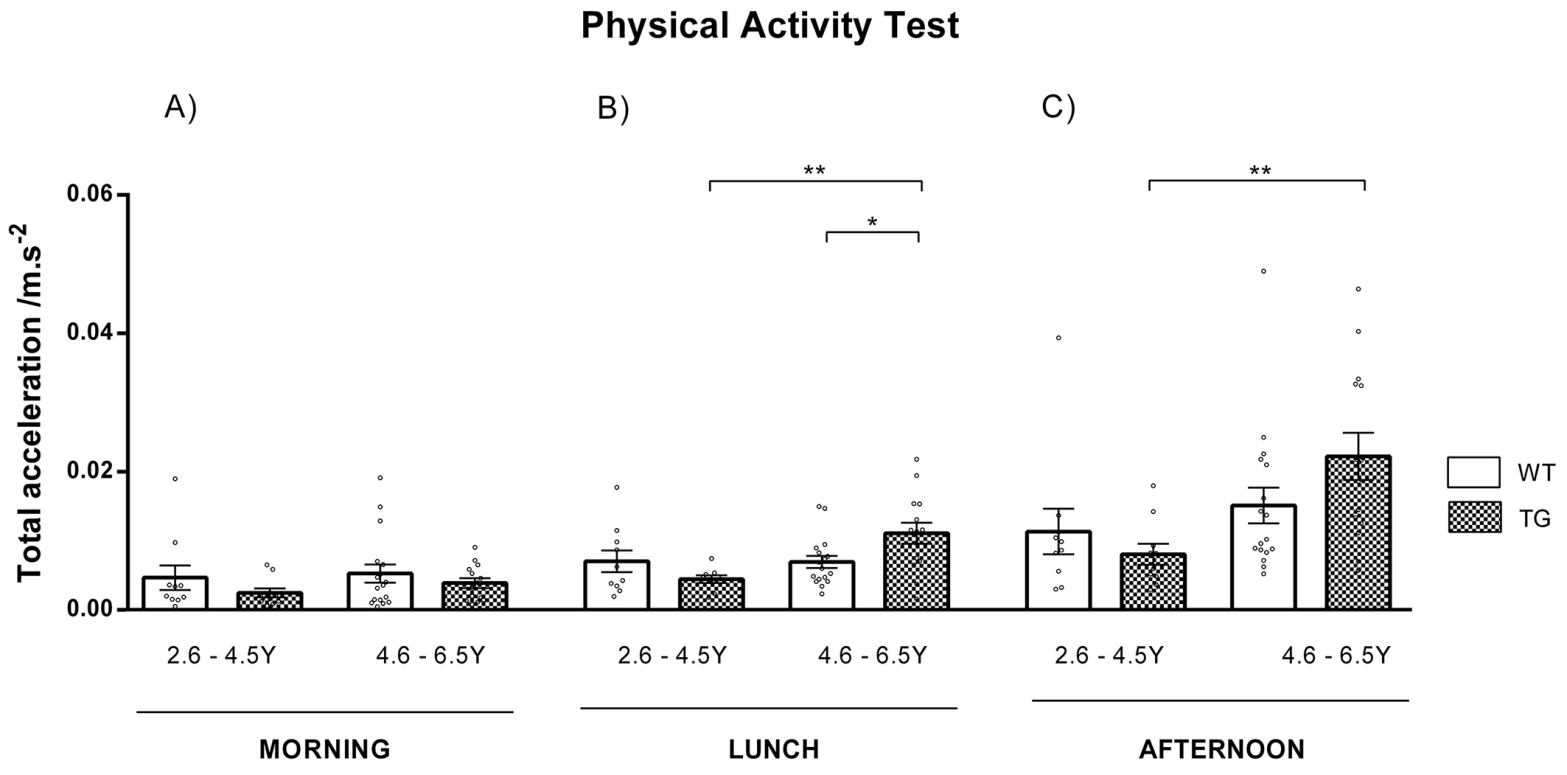
**Altered physical activity.** (A) In TgHD animals, physical activity was lower in the morning than in other periods. (B) During the lunch period, 2.6- to 4.5-year-old TgHD animals had comparable physical activity to that of their WT controls. The activity of 4.6- to 6.5-year-old TgHD animals was significantly (*P*=0.026) higher than that of the WT controls. Activity significantly (*P*=0.003) increased with age in TgHD animals. (C) In the afternoon period, 2.6- to 4.5-year-old TgHD animals had decreased physical activity, whereas 4.6- to 6.5-year-old TgHD animals had increased physical activity, in comparison to that of their WT controls, although these differences were not statistically significant. Significantly increased (*P*=0.002) physical activity was observed with aging in TgHD animals. WT boars 2.6-4.5 years *n*=7, WT boars 4.6-6.5 years *n*=17, TgHD boars 2.6-4.5 years *n*=10, TgHD boars 4.6-6.5 years *n*=14. *P≤0.05, **P≤0.01.

People with HD tend to be less physically active in the morning and more active in the afternoon and evening. This might be caused by sleep disturbances and shifted circadian rhythm in HD individuals (Herzog-Krzywoszanska and Krzywoszanski, 2019). The physical activity of minipigs was investigated by telemetric assessment. TgHD boars were monitored during a 1-week period in six different sessions. Physical activity was evaluated over three periods: morning (02:30-04:30, before morning feeding), lunch (09:40-12:00, no external activity) and afternoon (14:50-15:50, before afternoon feeding). Total acceleration computed by a telemetric system was averaged in 10-min intervals. Evaluation of the morning data (Fig. 5A) revealed decreased activity in TgHD boars compared to WT controls, although these differences were non-significant. During the lunch period (Fig. 5B), the physical activity of 2.6- to 4.5-year-old TgHD boars was comparable to that of WT animals, whereas the physical activity of 4.6- to 6.5-year-old TgHD animals was significantly (*P*=0.026) increased in comparison to that of WT controls. The physical activity of 4.6- to 6.5-year-old TgHD animals was also significantly higher (*P*=0.003) than that of 2.6- to 4.5-year-old TgHD animals. Similarly, in the afternoon period (Fig. 5C), physical activity was similar between younger TgHD and WT animals (aged 2.6-4.5 years) and increased in the older animals (aged 4.6-6.5 years). During the lunch and afternoon periods, the physical activity of the TgHD animals significantly (*P*=0.003 and *P*=0.002, respectively) increased with age.

## DISCUSSION

Transgenic minipigs encoding truncated human mHTT (Baxa et al., 2013) were monitored in a longitudinal phenotypic study utilizing motor, cognitive and behavioral tests. All of the examined animals from F0-F3 generations carried one copy of the mHTT gene with 124 mixed CAG/CAA repeats incorporated in chromosome 1. Previously, no significant difference was observed between TgHD and WT sows up to 40 months of age (Schuldenzucker et al., 2017). A significant decline in the ability to perform the tunnel test, and a general tendency (non-significant) for reduced accomplishment in other motor, behavioral and cognitive tests, was detected in a mixed group of TgHD males and females at the age of 48 months (Askeland et al., 2018).

In this study, walking, hurdle, pull-back, tongue, skittles, cover pan, balance beam, seesaw and physical activity tests were performed in a larger group of animals at the age of 4-5.9 and 6-7.9 years, and the impact of sex was also taken into account. An overview of the test outcomes is provided in Table 1. The data obtained from the 4 subsequent years enabled us to compare the progress of the observed changes with the age of animals. Simultaneously, it enabled us to enlarge the number of animals per age group as the individual animals were tested at the age of x, x+1, x+2 and x+3 years.

**Table 1.**
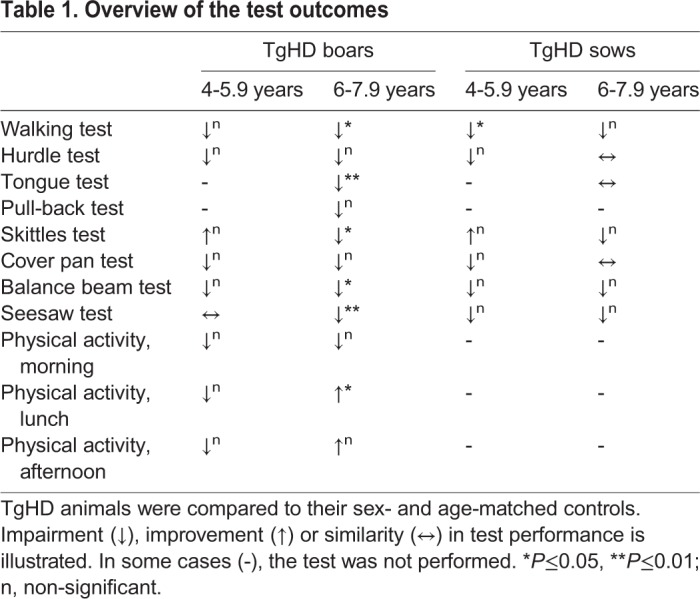
**Overview of the test outcomes**

Stricken gait problems to maintain upright posture and impaired balance are obvious motor symptoms in HD patients (Rüb et al., 2013). Contrary to humans, animal models, excluding non-human primates, are tetrapods; therefore, they are more stable in motion. Nevertheless, walking and pull-back tests showed motor impairment in the TgHD minipigs. Worsening performance in the walking test (significantly at the age of 6-7.9 years) was evident in TgHD boars, but was not so obvious in TgHD sows. We suppose that this difference is caused by the body constitution of males and females. Sows are shorter and wider, whereas boars are taller, thus the center of gravity provides an advantage to females. Moreover, the center of gravity in boars is more rostral, which could make them more vulnerable to losing balance in the hind legs. Significant decline in walking was observed in TgHD sows at 4-5.9 years of age. We assume that this relates to the lower animal body mass index (ABMI, calculated as a ratio of an animal's weight, height and length) of TgHD sows at 6-7 years compared to that of WT sows (Ardan et al., 2020). Weight loss is a prominent feature of HD and it could facilitate the movement process in TgHD females. Similar to HD patients, TgHD boars showed disturbed balance and longer corrective response to an unexpected push. However, the animals were able to learn and predict the coming push from obtained experience. This was shown by their increased ability to maintain stability when the test was repeated (data not shown). To our knowledge, these are the first impairments in motor phenotype with adult-age onset described in a large animal model of HD.

Concerning fine motor skills, glossomotography revealed impairment of tongue protrusion in HD patients (Reilmann et al., 2010). In the tongue test, TgHD boars reached treats from shallower holes than their WT counterparts, but this difference was not significant; however, the number of holes they reached was significantly lower than that of the WT males. We suppose that the deepest-reached-hole test showed the possible changes in fine motor skills, whereas the number-of-reached-treats test reflected the willingness/interest of the animals to perform the test. A different situation was observed in TgHD sows. The number of reached holes, and their depth, was similar to that of WT controls. Similarly, in a previous study, no significant changes were detected in TgHD sows up to 40 months of age (Schuldenzucker et al., 2017). We suppose that this could be an effect of housing TgHD and WT sows in shared pens, in which the dominant WT sow(s) could eat more food than the TgHD sows, causing the TgHD sows to feel hungrier and therefore more motivated to reach for the treat. This effect may be further supported by hyperphagia caused by attenuation of the frontal lobe (cognitive and behavioral deterioration) commonly observed in HD patients (Trejo et al., 2004).

Similar results were observed in skittle and cover pan tests. These tests were assessed to investigate cognitive decline (Dumas et al., 2013). Age-dependent diminution in performance in the skittles tests was observed in TgHD boars. Tests investigating cognition were established in sheep, non-human primates and minipigs. Choice discrimination tests reflecting reversal learning have been used in WT sheep (McBride et al., 2016) and TgHD minipigs (Schuldenzucker et al., 2017). No significant differences in a choice discrimination test or in a dominance test, which was used to test dominant/aggressive behavior in TgHD sows up to 40 months of age (Schuldenzucker et al., 2017), were observed.

Balance beam and seesaw tests mirror response to stress stimuli and recollection of memories, representing the tied connection between cognition and emotions (Marino and Colvin, 2015). Stress-induced tests revealed a continuous decline in TgHD animals with age, compared to their WT counterparts, and this difference was significant in 6- to 7.9-year-old TgHD boars. The two tests required courage to succeed in the task. Females and TgHD boars showed more fear to step on/pass the balance beam/seesaw. Thus, the observed impairment was a combined result of the abilities, characters and emotions of the individual animals.

Perspicuous sex-related differences were observed in all tests performed in this study. Although TgHD boars and TgHD sows both had reduced performance in the tests compared to their WT counterparts, TgHD boars showed a significant decrease in performance in the walking, balance, seesaw and skittles tests at the age of 6-7.9 years, but sows did not. The boars demonstrated more courage to complete the tasks, whereas the sows were generally fatter and more hesitant by nature, presumably leading to the results achieved. Sex commonly modulates human behavior, including many aspects of human brain development (Cahill, 2014). The pattern of structural brain changes associated with huntingtin is strikingly different between men and women (Lee et al., 2017), which could explain the observed differences in test performances among TgHD boars and sows.

Telemetric evaluation of physical activity revealed that TgHD boars of younger ages (2.6-4.5 years) had lower activity throughout the day than their WT controls, although this difference was non-significant. However, from the age of 4.6 years, physical activity was reduced in the morning period, and increased during the lunch and afternoon periods. A significant increase in physical activity was observed in TgHD boars with age, which could be associated with the manifestation of disease progression. Similarly, decreased morning activity and increased physical activity in the later hours of the day have been observed in HD patients. This feature relates to their impaired circadian organization (Herzog-Krzywoszanska and Krzywoszanski, 2019; Kudo et al., 2011; Morton et al., 2005).

Recently, a new HD knock-in porcine model with 150 glutamines in the polyQ tract was generated using a CRISPR/Cas9 approach (Yan et al., 2018). Walking abnormalities were observed at the age of 5 months. Brain degeneration was manifested by the loss of DARPP32-positive cells, activated microglia, increased level of GFAP and demyelination. The same markers of neurodegeneration were detected in a TgHD minipig model but much later, starting at 16-70 months of age (Baxa et al., 2013; Vidinská et al., 2018; Ardan et al., 2020). Enlarged ventricles were revealed in an HD knock-in model at 5 months of age. Similarly, magnetic resonance imaging (MRI) data showed expansion of the lateral ventricles of TgHD minipigs at 6-7 years, but only three animals were assessed (J. Klima, J.J. and S.J. et al., unpublished). According to differences in HD manifestation between TgHD and HD knock-in models, the models may have pros and cons regarding their use, depending on the intended studies. The HD knock-in model could be used for studies on HD pathology and rescue effects after treatment, whereas the TgHD model is suitable for testing longitudinal HD therapeutic treatments due to the long premanifestation stage (Evers et al., 2018).

In conclusion, our longitudinal study has shown gradual progression in TgHD minipigs. The test outcomes correlate with disease progression and could be applied in preclinical tests, particularly in ongoing longitudinal AAV5-miHTT (microRNA targeting HTT sequence) studies in minipigs after they reach the age of phenotype manifestation. Based on promising results from the first minipig studies (Evers et al., 2018), the Food and Drug Administration and the European Medicines Evaluation Agency approved this approach for phase-I and -II clinical trials. If the here established methods and measures could postpone phenotype development in TgHD minipigs in ongoing longitudinal AAV5-miHTT experiments, they could – together with the clinical data – shorten the time for patients to receive a cure.

## MATERIALS AND METHODS

### Animals

Transgenic minipigs (Sus scrofa domesticus, Linnaeus) expressing one copy of N-truncated human mutated huntingtin with 124 glutamines in polyQ sequence (Baxa et al., 2013) and their WT controls were used in this study. The individual animals were subjected to tests over the 4 subsequent years (2015-2018). Eight TgHD (four males, four females) and ten WT control (five males, five females) animals at the age of 4-7.9 years were monitored in motor, cognitive and behavioral tests (Table S1). Five animals died within the study (one WT boar, aged 6 years; one TgHD boar, aged 6 years; three TgHD sows, aged 6, 7 and 8 years). Six TgHD minipigs and their six WT siblings at the age of 2.6-6.5 years were used for the telemetry study (Table S1). See Table S1 for further details on the animals used. All experiments were carried out according to the guidelines for the care and use of experimental animals and approved by the Resort Professional Commission of the Czech Academy of Sciences for Approval of Projects of Experiments on Animals (approved protocol no. 53/2015).

### Walking test

Changes in gait were observed by walking the animal on a dry, flat floor. Gait was video-recorded at 6-month intervals. Scoring was as follows: 5 points for no visible gait problem and fluent walking; 4 points for slightly uneven weight bearing on one or more legs; 3 points for obvious deviation in weight bearing on one or more legs, with clear difficulties in walking; 2 points for lowering of the hind quarters close to the ground, placement of hind legs under the body; 1 point if the pig is unable to move; 0 points if the animal refused to perform the test (Askeland et al., 2018).

### Hurdle test

**Fig. 6.**
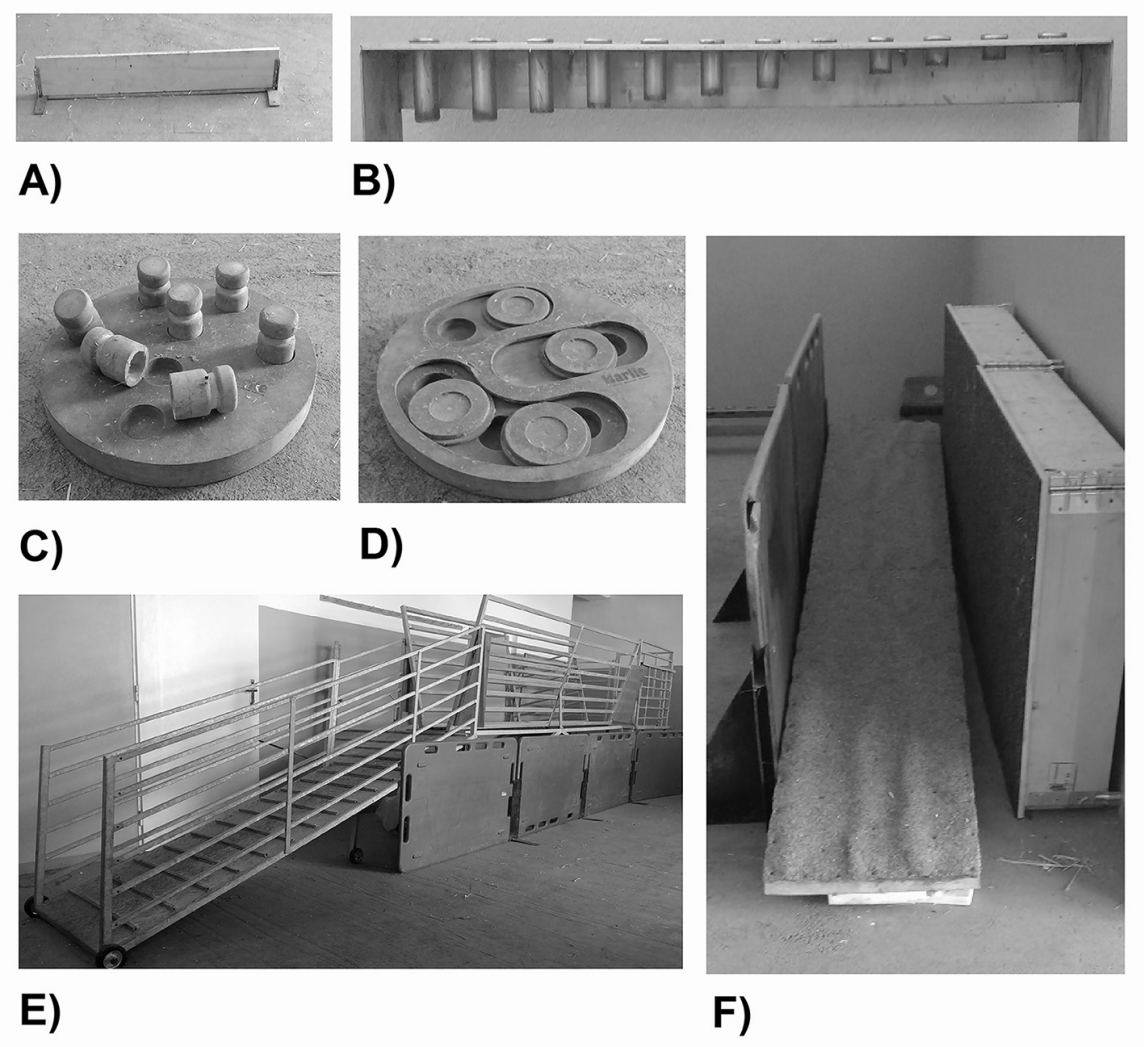
**Equipment for motor, cognitive and behavioral studies.** (A) Hurdle. (B) Tongue board. (C) Skittles pan. (D) Cover pan. (E) Balance beam. (F) Seesaw.

In the hurdle test (Fig. 6A), the animals were expected to cross the hurdle (height 15 cm, width 100 cm). Scoring was as follows: 5 points for crossing the hurdle without touching; 4 points for crossing the hurdle with one leg touching; 3 points for crossing the hurdle with two legs touching; 2 points for crossing the hurdle with three legs touching; 1 point for crossing the hurdle with four legs touching; 0 points if animal refused to perform the test (Askeland et al., 2018).

### Pull-back test

The animals were allowed to walk on a dry, flat floor and an unexpected shove was performed to the haunch area. Stability and reaction to the sudden external force were evaluated.

### Tongue test

The tongue test (Fig. 6B) was performed similarly as described previously (Schramke et al., 2016; Schuldenzucker et al., 2017). Briefly, a board containing 12 holes with stepwise increasing depth from 1 cm to 6.5 cm was used. Pigs were expected to pick up as many treats (biscuits) as possible from a board. The holes were marked from 1 (shallowest) to 12 (deepest). Two parameters were analyzed and the scoring was as follows: number of reached treats, 1 point for each one hole with an eaten treat; deepest hole, the score was the mark of the deepest hole from which the treat was reached.

### Skittles test

In the skittles test (Fig. 6C), the animals were expected to flip as many skittles as possible. Scoring was as follows: 1 point for each tumbled skittle (1-7 points); 0 points if the animal refused to perform the test.

### Cover pan test

In the cover pan test (Fig. 6D), treats were hidden under six movable/sliding covers. Animals were expected to move the cover and reach the treat under it. Scoring was as follows: 1 point for each cover (1-6 points); 0 points if the animal refused to perform the test.

### Balance beam test

The balance beam test (Fig. 6E), consists of a 2.5-m inclined plane, a 3.0-m beam and an extended plane (1.15×1.3 m). The animal was expected to step up to an inclined plane, cross the beam, turn back in the extended part and return back down. Scoring was as follows: 5 points for passing across the whole beam, turning in the extended part and going back down; 4 points for passing across the whole beam, not turning in the extended part and crawfishing back down; 3 points for stepping on (any part of) the beam; 2 points for stepping on the inclined plane with all four legs; 1 point for stepping on the inclined plane with two forelegs; 0 points if the animal refused to perform the test (Askeland et al., 2018).

### Seesaw test

During the seesaw test (Fig. 6F), the pig was expected to pass over the seesaw (3.0 m length, 0.4 m width). Scoring: 5 points for passing across the whole seesaw; 4 points for passing to the equilibrium position, crawfishing back down; 3 points for stepping onto and walking on the seesaw with all four legs (the equilibrium position not reached); 2 points for stepping on the seesaw and crawfishing back; 1 point for stepping on the seesaw with only two forelegs; 0 points if the animal refused to perform the test (Askeland et al., 2018).

### Evaluation of motor, cognitive and behavioral tests

A battery of motor, cognitive and behavioral tests was conducted three to five times per year. The mean data from all the measurements collected within 1 year were calculated for each individual animal. The groups of animals were selected according to their genotype, sex and age. Each data point in the graph represents one individual animal at a defined age. Individual animals were tested within the 4 subsequent years, thus data were collected from their ages at x, x+1, x+2 and x+3 years.

### Telemetric monitoring of physical activity

TgHD boars were monitored for 1 week in each session. Physical activity was evaluated over three periods: morning (02:30-04:30, before morning feeding), lunch (09:40-12:00, no external activity) and afternoon (14:50-15:50, before afternoon feeding). Telemetric system rodentPACK2 obtained from emka TECHNOLOGIES (France) was used in the experiments. The telemetric system consisted of small transmitters and central receivers. Every transmitter could acquire x, y, z and total acceleration. Data were sampled at a frequency of 100 Hz with resolution ±2 g. Minipig boars wore collars with transmitters during the experiments (Pokorný et al., 2015). A PC collected and stored data from receivers using iox2 software from emka TECHNOLOGIES. All events during the measurement sessions were recorded, e.g. time of feeding, cleaning, veterinary and technician intervention, or other activities. The first session including no disturbing activities was used for statistical analysis. The early-morning period (just before staff come after the night, before morning feeding) was analyzed. This period is hypothetically daytime with minimal external influences. Total acceleration [gravity acceleration (g)], computed by the telemetric system and representing the physical activity of the animal, was processed by our scripts for the open-source tool SciLab. The total physical activity was sampled at 100 Hz and averaged over 10 min. Generated physical activity (mean from 10 min) from the TgHD and WT boars was consequently averaged and used for statistical analysis.

### Evaluation of telemetric monitoring of physical activity

Physical activity was measured in six sessions (September 2015, December 2016, March 2017, September 2017, December 2017 and September 2018). The mean data from all measurements collected within 1 year were calculated for each individual animal. The groups of animals were selected according to their genotype and age. Each data point in the graph represents one individual animal at a defined age. Individual animals were tested within the 4 subsequent years, thus the data were collected from their ages at x, x+1, x+2 and x+3 years.

### Statistics

Evaluators of all of the tests were blinded for animal genotype. All statistical tests were carried out in GraphPad Prism. Calculation of statistical significance was computed using Student's *t*-test and Mann–Whitney test. All figures show the mean results and error bars represent s.e.m. Significance was set at *P*≤0.05 (*P≤0.05, **P≤0.01).

This article is part of a special collection ‘A Guide to Using Neuromuscular Disease Models for Basic and Preclinical Studies’, which was launched in a dedicated issue guest edited by Annemieke Aartsma-Rus, Maaike van Putten and James Dowling. See related articles in this collection at http://dmm.biologists.org/collection/neuromuscular.

## Supplementary Material

Supplementary information
