# Supplementary material for: Longitudinal study revealing motor, cognitive and behavioral decline in a transgenic minipig model of Huntington's disease
Source: Dis Model Mech. 2019 Dec 12;13(2):dmm041293. doi: 10.1242/dmm.041293 (PMC6918771; doi:10.1242/dmm.041293)
Supplement: Supplementary information [file dmm-13-041293-s1.pdf]

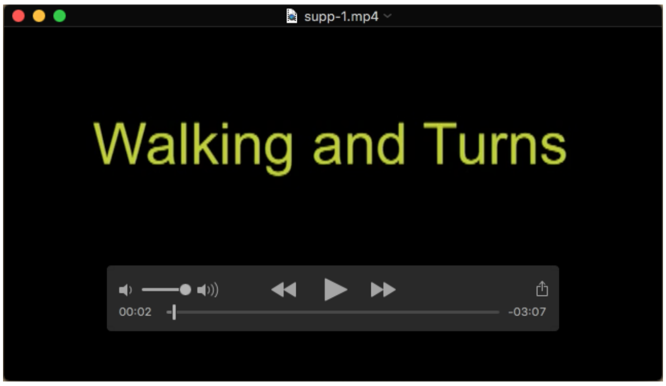

Movie 1. Walking test.

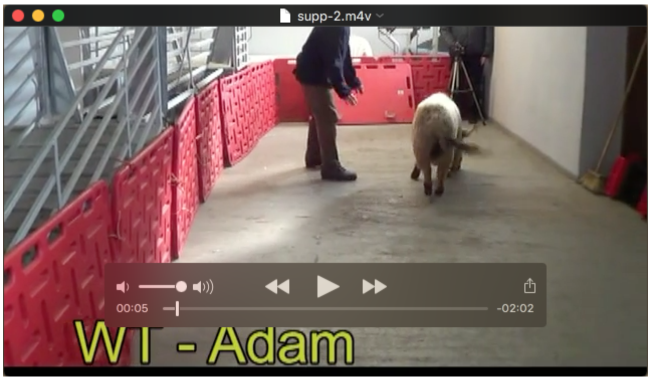

Movie 2. Pull back test.

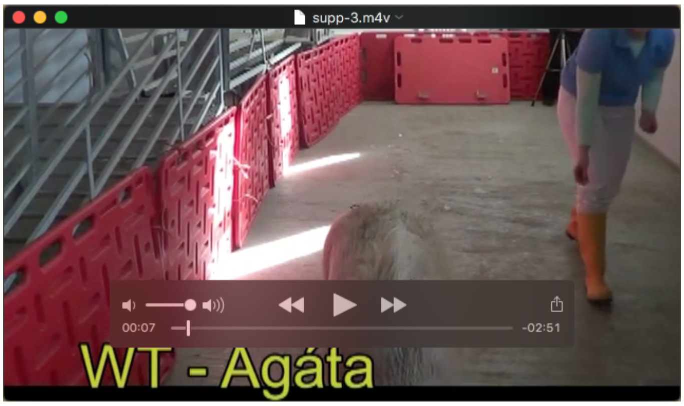

Movie. 3. Balance beam test.

Table S1. List of animals in longitudinal study.

Motor, Cognitive and Behavioral studies						
	Animal	Date of Birth	Generation	Number of CAG in mtHTT gene	Number of mtHTT copies	Date of death
WT ♂	F630	25-Nov-08	—			
	F808	05-Jul-09	F0			
	G292	28-Sep-10	—			
	K 151	16-Apr-11	F2			22-Aug-17
	K 353	22/-Sep-11	F2			
TG ♂	G117	29-Apr-10	F1	124	1	17-May-17
	G118	29-Apr-10	F1	124	1	
	K 150	16-Apr-11	F2	124	1	01-Jun-17
	K 152	16-Apr-11	F2	124	1	
WT ♀	G122	29-Apr-10	F1			
	K 66	26-Feb-11	F2			
	K153	16-Apr-11	F2			
	K155	16-Apr-11	F2			
	K188	27-Jul-11	F0			
TG ♀	F807	5-Jul-09	F0	124	1	28-Mar-17
	K 65	26-Feb-11	F2	124	1	
	K156	16-Apr-11	F2	124	1	
	K169	6-May-11	F2	124	1	
	K217	28-Jun-11	F2	124	1	13-Mar-17

Telemetry study of Physical Activity						
	Animal	Date of Birth	Generation	Number of CAG in mtHTT gene	Number of mtHTT copies	Date of death
WT ♂	L149	1-Jul-12	F3			
	L156	6-Aug-12	F3			
	L221	1-Oct-12	F3			
	L222	3-Oct-12	F3			
	L232	9-Oct-12	F3			
	L346	27-Feb-13	F3			
TG ♂	L218	1-Oct-12	F3	124	1	
	L219	1-Oct-12	F3	124	1	
	L223	3-Oct-12	F3	124	1	
	L272	10-Dec-12	F3	124	1	
	L292	26-Dec-12	F3	124	1	
	L345	27-Feb-13	F3	124	1	

Disease Models & Mechanisms • Supplementary information
